# Supplementary material for: Patterns of Intron Gain and Loss in Fungi
Source: PLoS Biol. 2004 Nov 30;2(12):e422. doi: 10.1371/journal.pbio.0020422 (PMC532390; doi:10.1371/journal.pbio.0020422)
Supplement: Table S1 — Also available at http://genes.mit.edu/NielsenEtAl/. (4.3 MB ZIP). [file pbio.0020422.st001.zip › NielsenEtAl/html/775.html]

AN0144.1.NCU01797.1.MG07012.1.FG04382.1


```
 CLUSTAL W (1.82) Multiple Sequence Alignments - Introns Inserted


Sequence 1: NCU01797.1	623 aa
Sequence 2: FG04382.1	612 aa
Sequence 3: AN0144.1	572 aa
Sequence 4: MG07012.1	547 aa
Alignment Length: 635 aa
Number Identitical Residues: 233 aa
Alignment Score (without introns) 12344


MG07012.1 	MSALKTTTNEKTSTGDKLRNFFKINNNRRRLPFGRS----KTGHEAPQAKSTRADDSDGA
NCU01797.1	MPSTKNA-NGEGHFPSRIKQFFRINSGSKDHKDRDA---HTTSSSHGGAPRADAKTPSGF
FG04382.1 	MPSQKNS-NGTGQVSHRLRNFFRMNTGGSTGSASSD---KEKDKDKEKNSASTTPDPSKP
AN0144.1  	MPTKKHP-NDSTHLPTKIKNFFRINSSSSTSSHQSSNHVNSSDRDTGSSTPVKHEPKSTF
          	*.: * . *       ::::**::*..         .    .. .            .  

MG07012.1 	QYT-------------THDDESQDANEPHSTPADS------EAARRIRRVK------SEP
NCU01797.1	RQSRFFS--VGRLRSTTVVSEGNPLDESMSPTAHANPYFAHQGQPGLRHHNDGSVPPSPP
FG04382.1 	RHTKFFSNTVGRLRAHTVASEGNQLEEAMSPTAHANPYFAHQGQPGLRHHNSDSVPPSPP
AN0144.1  	RQSRFLP-MIGRNRSATVASEGNPLDEAISPTATANPYFVHQGQPALQHRNDGSIPSSPP
          	: :   .   .   : *  .*.:  :*. *..* :..    :.   ::: :..: ..* *

MG07012.1 	SQLGLIFGYLSGG---RPATSGRGKEKTLLANKNASAVGLVGTSSSTPTPG---PSSTGG
NCU01797.1	DTPSLKVDGPEGS---QQPTAATKEELARKLRRVASAPNAQGLFSKGQGNGDRPATAELS
FG04382.1 	DTPSLKVHGPDGTPATDQTTSETKEELARRLRRVASAPNAQGLFSKNDPNNERPATAELG
AN0144.1  	DTPELQVDGVSAA---EQATTANKEELARKLRRVASAPNAQGLFTATDSDG-RPQTAELG
          	.   * .   ..      .*:   :* :   .: *** .  *  :     .  . ::  .

MG07012.1 	GECVIPSAGG----------------EIVVVPTPNAATPPHGGPKQ--KLVAYHRTYSSN
NCU01797.1	KEPLEESKDS---NTVGFAEQKPNNDSSTSLAAPDADGLGALPPPIRQSPLAFRRTYSSN
FG04382.1 	KDPLLTSSGA---LGLIEPAKTPQNEEMPTHANPE-DILAALPAPN--MSMAFRRTYSSN
AN0144.1  	KEPLVHSGAAGSPVGLVEVSSNTDQDGNLTVPQPGSGKLPAVTQVR-TSSGGFRRTYSSN
          	 : :  *  ..:.       ......     . * :               .::******

MG07012.1 	SIKKGPVRVGPEDFEKLKLIGKGDVGKVYLVREKKRDKLFALKM1LDKDEMIRRNKIKRT
NCU01797.1	SIKVRNVEVGPQSFDKIKLIGKGDVGKVYLVKEKKSGRLYAMKV1LSKKEMIKRNKIKRA
FG04382.1 	SIKVRNVEVSPSSFDKIKLIGKGDVGKVYLVREKKSSRLYAMKI1LSKKEMIKRNKIKRA
AN0144.1  	SIKVRNVEVGPASFDKIKLIGKGDVGKVYLVREKKSTRLYAMKV1LSKKEMIKRNKIKRA
          	***   *.*.* .*:*:**************:***  :*:*:*: *.*.***:******:

MG07012.1 	VTEQEILQVMNHPFIVSLHHSFQSQKYLYLCMEYCSGGEFFRA~LQSRPGKCISEEAARF
NCU01797.1	LAEQEILATSNHPFIVTLYHSFQSEDYLYLCMEYCSGGEFFRA~LQTRPGKCIPEDDARF
FG04382.1 	LAEQEILATSNHPFIVTLYHSFQSEDYLYLCMEYCSGGEFFRA~LQTRPGKCIPEEDARF
AN0144.1  	LAEQEILATSNHPFIVTLYHSFQSEDYLYLCMEYCSGGEFFRA1LQTRPGKCISEDAARF
          	::***** . ******:*:*****:.***************** **:******.*: ***

MG07012.1 	YIAEVISALEYLHMNGLIYRDLKPEN1ILLHESGHIMLSDFDLSKISGQDGMPTMIVGRN
NCU01797.1	YAAEVTAALEYLHLMGFIYRDLKPEN1ILLHQSGHIMLSDFDLSKQSDPGGKPTMIIGKN
FG04382.1 	YAAEVTAALEYLHLMGFIYRDLKPEN1ILLHQSGHIMLSDFDLSKQSDPGGKPTMIVGKN
AN0144.1  	YAAEVTAALEYLHLMGFIYRDLKPEN1ILLHQSGHIMLSDFDLSKQSGPGGAPTMIPARS
          	* *** :******: *:********* ****:************* *. .* **** .:.

MG07012.1 	GTTT----MLDTRSCLEGYRTNSFVGTEEYIAPEVIKGHGHSVAVDWWTVGILSFEMIYG
NCU01797.1	GTSTSSLPTIDTKSCIANFRTNSFVGTEEYIAPEVIKGSGHTSAVDWWTLGILIYEMLYG
FG04382.1 	GARTDALPTIDTRSCIANFRTNSFVGTEEYIAPEVIKGSGHTSAVDWWTLGILIYEMLYG
AN0144.1  	GNSNTSLPTIDTKSCIADFRTNSFVGTEEYIAPEVIKGCGHTSAVDWWTLGILIYEMLYG
          	*  . : . :**:**: .:******************* **: ******:*** :**:**

MG07012.1 	VTPFKGKNRNATFANVMREDVHFPDDPELSP2MG-~--KSFIR~KCLIKDEN~RRLGAIA
NCU01797.1	TTPFKGKNRNATFANILREDIPFPDHAGAPQ~ISN2LCKSLIR~KLLIKDEN~RRLGARA
FG04382.1 	TTPFKGKNRNATFANILREDIPFPDHAGAPQ~IS-~------K2KLLIKDEN~RRLGARA
AN0144.1  	TTPFKGKNRNATFGNILRDEVPFPEQGGAQQ~IS-~-------~KYVITEFC2R------
          	.************.*::*::: **:.      :.          * :*.:   *      

MG07012.1 	GAAELKRHPWFRTTQWALLRHLTPPIIPDQG---PDGNFRTLKDSQSVDIGG--------
NCU01797.1	GASDIKTHPFFRTTQWALIRHMKPPIVPNQGRGIDTLNFRNVKESESVDISGSRQMGLKG
FG04382.1 	GASDIKAHPFFRTTQWALIRHMKPPIVPHAGRGVDTVNFRNVKESESVDLSGSRAMNLKG
AN0144.1  	--------------------------------GNETVNFRNVKESASVDIGEKNPSKMKG
          	                                .    ***.:*:* ***:. .     ..

MG07012.1 	-------ATRKVADDDPFSAFSSVTLHYGDDK----------
NCU01797.1	EPLESGMVTPGENAVDPFEEFNSVTLHHDGDEEYHSDAYEKR
FG04382.1 	VPLDSGLATPGGEIADPFLEFNSVTLHHDGDDDHHR------
AN0144.1  	VPLNSGLATPNGEIADPFEEFNSVTLHHDGDF----------
          	 . .:. .*      ***  *.*****:..*
```
